# Genomic Identification and Expression Analysis of the Cathelicidin Gene Family of the Forest Musk Deer

**DOI:** 10.3390/ani9080481

**Published:** 2019-07-24

**Authors:** Long Zhang, Hang Jie, Yingping Xiao, Caiquan Zhou, Wentao Lyu, Wenke Bai

**Affiliations:** 1 Institute of Ecology, Key Laboratory of Southwest China Wildlife Resources Conservation (Ministry of Education), China West Normal University, Nanchong 637009, China; 2 Sichuan Wildlife Rehabilitation and Breeding Research Center, China West Normal University, Nanchong 637009, China; 3 Chongqing Engineering Technology Research Center for GAP of Genuine Medicinal Materials, Chongqing Institute of Medicinal Plant Cultivation, Nanchuan 408435, China; 4 State Key Laboratory for Quality and Safety of Agro-products (in prepared), Institute of Quality and Standard for Agro-Products, Zhejiang Academy of Agricultural Sciences, Hangzhou 310021, China

**Keywords:** cathelicidin, forest musk deer, phylogenetic analysis, expression pattern

## Abstract

**Simple Summary:**

Cathelicidins are a group of host defense peptides in vertebrates with both antimicrobial and immunomodulatory activities. In the present study, we identified the entire repertoire of the cathelicidin gene family from the forest musk deer genome. Sequence comparison, phylogenetic topology, and gene and genomic organizations collectively suggest that all cathelicidin genes have already been fixed in the genome of forest musk deer before the split of moschidae and bovidae, while independent pseudogenization events have occurred after species divergence. In addition, real-time PCR analysis suggested that all functional cathelicidins play important roles in the immune system. The results of this study will be helpful for further evolutionary and functional studies.

**Abstract:**

The forest musk deer (*Moschus berezovskii*) is a small-sized artiodactyl species famous for the musk secreted by adult males. In the captive population, this species is under the threat of infection diseases, which greatly limits the increase of individual numbers. In the present study, we computationally analyzed the repertoire of the cathelicidin (CATHL) family from the genome of forest musk deer and investigated their expression pattern by real-time PCR. Our results showed that the entire genome of forest musk deer encodes eight cathelicidins, including six functional genes and two pseudogenes. Phylogenetic analyses further revealed that all forest musk deer cathelicidin members have emerged before the split of the forest musk deer and cattle and that forest musk deer *CATHL3L2* and *CATHL9* are orthologous with two cattle pseudogenes. In addition, the gene expression results showed that the six functional genes are not only abundantly expressed in the spleen and lung, but are also differently expressed in response to abscesses, which suggests that forest musk deer cathelicidins may be involved in infections. Taken together, identification and characterization of the forest musk deer cathelicidins provide fundamental data for further investigating their evolutionary process and biological functions.

## 1. Introduction

The forest musk deer (*Moschus berezovskii*), also known as the dwarf musk deer, is a small-sized artiodactyl species that is mainly distributed in the forested and mountainous regions of South-West Asia [1]. As a result of overhunting and habitat destruction in the past, the size of the wild forest musk deer population has been sharply reduced. This species has been listed as a first-class key species of wildlife in the Chinese Wild Animal Protection Law and is listed in the Appendices of Convention on International Trade in Endangered Species of Wild Fauna and Flora, as well as on the International Union for Conservation of Nature’s Red List of Threatened Species [1,2,3]. Since the 1950s, musk deer farming has been widely used to increase the individual numbers of musk deer and provide sustainable musk resources in China [4]. However, restricted by insufficient space and other animals’ natural behavioral needs in the artificial environment, disease is becoming a bottleneck that prevents the increase of captive populations [5,6]. Consequently, understanding immunity, including the genetic information of immune genes, is urgently needed to enhance the disease resistance of forest musk deer.

Host defense peptides (HDPs) comprise a large and diverse group of cationic amphipathic short peptides in virtually all forms of life [7,8,9]. With potent antimicrobial and immunomodulatory activities and many additional functions, HDPs play multifunctional roles in both innate and adaptive immunity [10]. Cathelicidins (CATHLs) are a major family of HDPs that have been found in a large range of species ranging from hagfish to human [11,12,13,14]. Cathelicidins consist of a short signal peptide, a highly conserved cathelin-like domain and a mature peptide usually consisting of fewer than 40 amino acids [15,16]. The number of cathelicidin genes differs greatly between species, with pig, chicken and cattle having multiple cathelicidin family members, whereas only one gene has been found in humans and mice [16,17,18,19]. In addition, the cathelicidin family components in many clades, particularly in cetartiodactyla, are largely diverse in mature peptide structure. For instance, the mature peptide of human cathelicidin is α-helical [18], while the cattle cathelicidin repertoire includes peptides with linear, β-turn and proline-rich structures [20]. 

Although numerous cathelicidin genes with high diversity have been reported in a large number of phylogenetically divergent species, no information is available regarding the forest musk deer. Fortunately, the recent completion of the musk deer genome [21] has provided an unprecedented opportunity to conduct a genome-wide screening of the forest musk deer cathelicidin gene repertoire. In this study, we identified eight cathelicidin genes from the genome sequences of forest musk deer and systematic analyzed their phylogenetic relationships. Furthermore, we examined the expression of musk deer cathelicidins in four different tissues and compared the mRNA abundance of the functional members in the liver and spleen between healthy musk deer and those who died of abscess disease. Our findings in this work will be helpful in understanding the immunity of the forest musk deer and provide fundamental information for further studies.

## 2. Materials and Methods 

### 2.1. Discovery of Cathelicidin Genes in the Musk Deer

To identify the entire repertoire of the cathelicidin gene family in the forest musk deer, a genomic identification strategy was conducted by a combination of the hidden Markov model (HMM) and the BLAST program, as we previously described [22]. Briefly, the hidden Markov model file of cathelicidin (accession number: PF00666.16) was downloaded from the Pfam database [23] and then used as a query to search against the predicted protein databases by using the HMMer (V3.1) program with the default settings. All potential hits with both E-values (full sequence and best 1 domain) <0.1 were then extracted and examined for the presence of the characteristic cathelin-like sequence. For every sequence identified, iterative TBLASTN [24,25] searches were conducted in the genome of musk deer (E-value < 10) until no new sequences were identified. The 4000-bp genomic sequences up- and downstream of each identified cathelin-like sequence were extracted from the genome for prediction of the full coding sequence with GeneWise [26] or GeneScan [27]. 

### 2.2. Collection of Tissues

The tissue samples, including the muscle from left hind leg, heart, lung, and spleen, were collected from three male captive musk deer died of natural disaster (earthquake of 20 April 2013), and three male captive musk deer died of abscess disease (mainly occurred in the lung). Tissues were cut into small pieces and immediately frozen in liquid nitrogen, and then stored at −80 °C until RNA extraction. These six musk deer ranged from six months to six years old and were raised on a farm at the Chongqing Institute of Medicinal Plant Cultivation. All experiments were approved by the ethics committees of the Chongqing Institute of Medicinal Plant Cultivation and were performed in accordance with the approved guidelines (DXY-S20160907) [28].

### 2.3. RNA Extraction and Real-Time PCR

Total RNA was extracted with TRIzol (Ambion) from all four tissues, RNA concentrations were measured by Nanodrop, and the first-strand cDNA was synthesized from 300 ng of total RNA using the PrimeScript^®^ RT reagent kit with gDNA eraser (Takara Bio Inc., Dalian, China) according to the manufacturer’s instructions. The integrity of RNA and absence of genomic DNA contamination of were verified as previously described [29,30]. Real-time PCR was conducted to examine the expression of genes, and the gene-specific primers were designed by Primer 5.0 software based on the predicted cathelicidin sequences and the conserved sequences in bovidae (Table 1). The relative expression levels of each cathelicidin gene in different tissues were analyzed using the 2^−ΔΔCT^ method [31], with the glyceraldehyde-3-phosphate dehydrogenase (GAPDH) gene being employed as the reference gene. Real-time PCR analysis of each sample was repeated in triplicate.

### 2.4. Multiple Sequence Alignment and Phylogenetic Analysis

All predicted amino acid sequences of the forest musk deer, as well as the reported amino acid sequences of cattle and sheep, were used for phylogenetic analysis. Sequences were aligned codon-to-codon using the MUSCLE program [32] and visualized with Boxshade (https://embnet.vital-it.ch/software/BOX_form.html). A neighbor-joining tree was constructed in Mega 7.0 [33] by calculating the *p*-distance among all sequences, and the reliability of each branch was tested by 1000 bootstrap replications.

### 2.5. Comparison of the CATHL3L2 C-Terminus of the Forest Musk Deer and Cattle

The DNA sequences of cattle CATHL3L2p C-terminus were retrieved from cattle genome (ARS-UCD1.2), and aligned with the corresponding region of the forest musk deer CATHL3L2 by Mega 7.0 [33].

### 2.6. Statistical Analysis

The expression levels of the cathelicidins between the tissues of healthy and purulent individuals were analyzed using unpaired Student’s *t*-test in GraphPad Prism 5 (GraphPad Software, La Jolla, CA, USA), *p* < 0.05 was considered significant between the healthy and purulent groups.

## 3. Results

### 3.1. Identification of the Forest Musk Deer Cathelicidin Genes

By using the HMMER program, a total of six sequences (scaffold41.3782, scaffold116.8830, scaffold204.11849, scaffold511.18163, scaffold511.18172, scaffold511.18173) were obtained from the predicted protein data of musk deer with the aforementioned settings. The subsequent confirmation workflows showed that scaffold116.8830 and scaffold204.11849 present secreted phosphoprotein 24 and cystatin domains, respectively, while scaffold511.18172 contains five cathelicidin domains. Therefore, the initial query file of the TBLASTN search contains the full amino acid sequences of scaffold 41.3782, scaffold 511.18163 and scaffold 511.18173, as well as the five sequence segments extracted from scaffold 511.18172, with each sequence containing one cathelicidin domain. In the end, nine genes, including eight cathelicidin genes and one neutrophilic granule protein (NGP) gene, were identified from the forest musk deer genome (Table S1). Subsequently, the eight cathelicidins were designated based on their orthologous relationships with their bovine counterparts.

**Figure 1:**
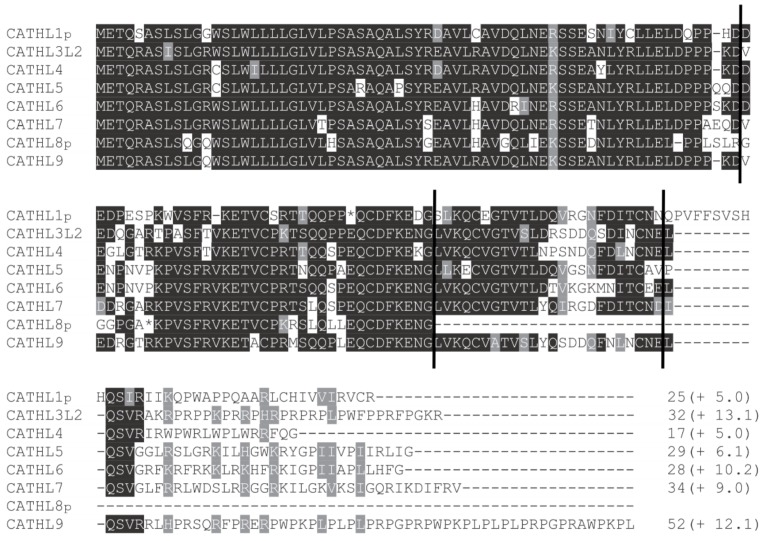
Multiple sequence alignment of forest musk deer cathelicidins. The positions of four exon boundaries are indicated by vertical lines, and conserved residues are shaded. The predicted C-terminal mature peptides are shown with the lengths and net charges (in parenthesis). The mature peptides of the forest musk deer cathelicidin genes were predicted based on the amino acid alignment of other cathelicidin genes, and the net charge of each mature peptide was estimated by using the on-line software at https://pepcalc.com/.

By aligning the deduced amino acid sequences of the eight forest musk deer cathelicidins (Figure 1), we observed a high level of conservation in the signal peptides and the cathelin-like domains but a large variation in the C-terminal mature peptides. Furthermore, two of the eight cathelicidin genes, termed *CATHL1p* and *CATHL8p*, were pseudogenes containing premature stop codons. Moreover, we noticed that *CATHL8p* was not an intact gene, which only contains the signal peptide and a part of the cathelin-like domain. The absence of the remaining parts of the *CATHL8p* gene could be caused by pseudogenization followed by sequence degeneration. Alternatively, the *CATHL8p* gene might have evolved from a partial gene copy event. 

In an attempt to determine the cationicity of forest musk deer cathelicidins, we predicted the net charge of their deduced mature peptides. As shown in Figure 1, all of these cathelicidin peptides were positively charged due to the presence of an excess number of cationic residues. The net charges of the mature peptides range from 5.0 to 13.1, implying that these molecules are able to interact with the negative charges located on the bacterial surface.

### 3.2. Phylogenetic Analysis of the Forest Musk Deer Cathelicidins

**Figure 2:**
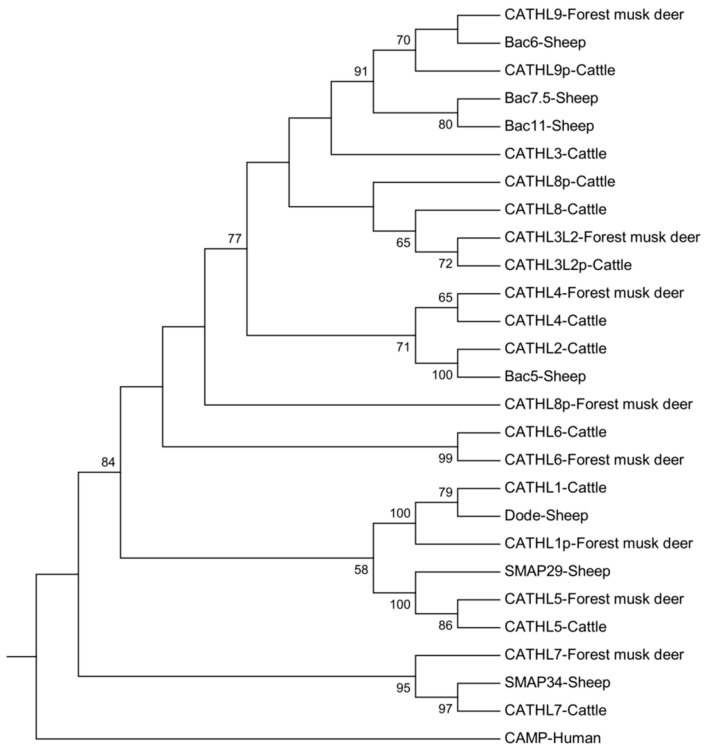
Phylogenetic relationships of the forest musk deer cathelicidins. Amino acid sequences of the forest musk deer cathelicidins were compared with sequences from other representative species. The phylogenetic tree was conducted by the neighbor-joining method with bootstrapping (1000 iterations). Only the bootstrap values higher than 50 are shown above branches.

To reveal the evolutionary relationships of the forest musk deer cathelicidins, a phylogenetic tree was constructed by using the neighbor-joining method together with the reported cathelicidin peptides from the sheep and cattle (File S1). As illustrated in Figure 2, the *CATHL1p*, *CATHL3L2*, *CATHL4-7*, and *CATHL9* of the forest musk deer have an orthologous gene in the cattle, which suggests that these genes existed before the split of the cattle and musk deer before 24.6 million years ago [34]. However, the *CATHL1p* in the forest musk deer is a pseudogene, suggesting that the pseudogenization of the forest musk deer *CATHL1* emerged after the species diverged. Interestingly, no sequence of the forest musk deer is located in the same subclade of the bovine *CATHL3*; instead, together with sheep *Bac6*, a cathelicidin gene of forest musk deer is clearly clustered with the cattle *CATHL9p* with a bootstrap value of 70. In addition, supported by a value of 72, the forest musk deer *CATHL3L2* also shares the same branch with a pseudogene termed cattle *CATHL3L2p*. Given that multiple paralogs of *CATHL3* exist in the genome of sheep and cattle, our results imply that gene duplication, diversification, pseudogenization, and even gene loss occurred in these three species. 

### 3.3. Comparison of the C-Terminus of the Forest Musk Deer CATHL3L2 and Cattle CATHL3L2p

**Figure 3:**
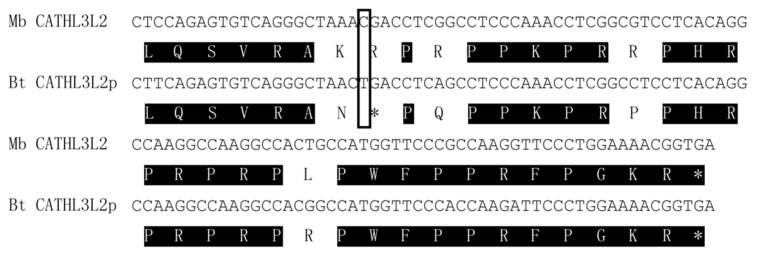
Sequence comparison of C-terminal sequences of *CATHL3L2* from the forest musk deer (Mb) cattle (Bt). Identical amino acids are shadowed, and the asterisk represents the termination codon. The nucleotide position that caused the pseudogenization of the cattle *CATHL3L2* was boxed.

Although the phylogenetic analysis suggested that forest musk deer *CATHL3L2* may be an ortholog of cattle *CATHL3L2p*, the amino acid comparison of the genes exhibited low similarity in the C-terminus. The previously deduced cattle *CATHL3L2p* seem to lack a few sequences in the mature peptide [19]. To confirm that the forest musk deer *CATHL3L2* is an ortholog of the cattle *CATHL3L2p*, we analyzed the C-terminal sequences of the cattle *CATHL3L2p*. As shown in Figure 3, a 28-amino-acid open reading frame was identified from the sequences immediately following the first stop codon of the cattle *CATHL3L2p*. Furthermore, the deduced amino acid sequences of this open reading frame are almost identical to the corresponding region of the forest musk deer *CATHL3L2*, which strongly indicates that the two cathelicidins are orthologous genes and that a previously unnoticed premature stop codon is also located in the putative mature peptide of the cattle *CATHL3L2p*.

### 3.4. Gene and Genome Organization of the Forest Musk Deer Cathelicidin Gene Family

To evaluate the gene structure of the forest musk deer cathelicidins, we compared the deduced amino acid sequences with the genomic sequence data. Similar to cathelicidin genes in other species [17,19], the coding sequence of each functional forest musk deer cathelicidin gene consists of four exons separated by three introns (Table 2), suggesting a high conservation of gene structure in cathelicidins.

**Figure 4:**
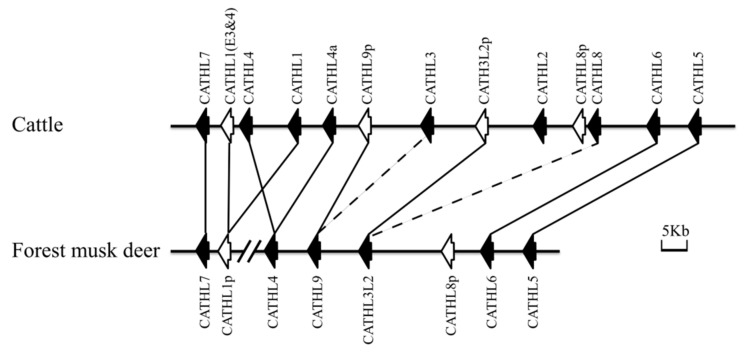
Genomic organization of the cathelicidin clusters in the cattle and forest musk deer. The position and transcriptional direction of each gene are represented by arrows. The solid arrows refer to functional genes, whereas open arrows refer to pseudogenes. A broken line in a gene cluster is an indication of a gap in the genomic DNA sequence. The orthologous genes are linked using solid lines, and paralogous genes are connected with dashed lines.

Coupled with the cattle, we strategically analyzed the genomic structure of the forest musk deer cathelicidins. As illustrated in Figure 4, the forest musk deer cathelicidins are clustered densely in the chromosomal region, although they are located in two different scaffolds. Additionally, we noticed that the gene order and orientation of all the forest musk deer cathelicidin genes, including the functional genes and pseudogenes, are highly conserved in the cattle. Together with the phylogenetic relationship, these observations suggested that both the functional genes and pseudogenes already existed in the forest musk deer before the divergence of the forest musk deer and cattle, while no allele comprising duplicate genes has been fixed in the forest musk deer genome since 24.6 million years ago [34].

### 3.5. Tissue Expression Pattern of Forest Musk Deer Cathelicidins

**Figure 5:**
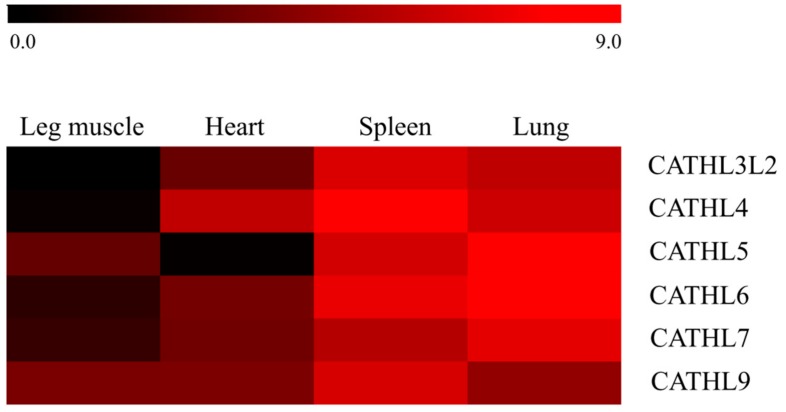
Relative expression levels of functional cathelicidin (CATHL) genes in forest musk deer. The expression levels of the forest musk deer cathelicidins were calculated relative to that of *CATHL3L2* using glyceraldehyde-3-phosphate dehydrogenase (GAPDH) as a reference gene. The color elements represent average log_2_ ratios of fold change from three samples.

As summarized in Figure 5, all six functional forest musk deer cathelicidins were ubiquitously expressed in all examined tissues from healthy animals, with the lowest expression level being observed in the leg muscle and relatively more abundant expression being detected in the other three tissues. The relatively higher mRNA levels in the spleen and lung indicated that cathelicidins might play critical roles in the immunity of forest musk deer. As expected, the expression of *CATHL1p* could not be detected in any of the tissues, which strongly suggests that *CATHL1p* is a pseudogene instead of a null allele in the forest musk deer (data not shown). At the same time, the detection of *CATHL3L2* and *CATHL9* within the cDNA of these tissues evidently suggests that they are functional genes in the forest musk deer.

**Figure 6:**
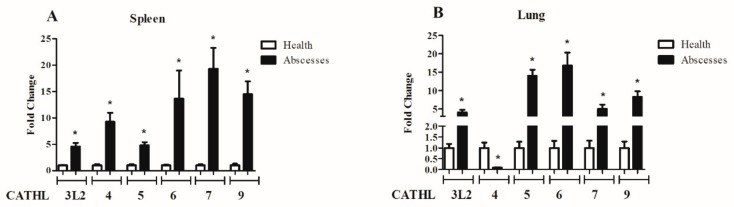
Comparison of the relative expression levels of cathelicidins in spleen (**A**) and lung (**B**) of healthy and purulent individuals. The relative gene expression was measured by real-time PCR using glyceraldehyde-3-phosphate dehydrogenase (GAPDH) as a reference gene. The bars represent the means ± standard error of mean (SEM). Differences between the healthy and purulent groups were determined by an unpaired Student’s two-tailed *t*-test, and * indicates *p* < 0.05.

We further compared the transcript abundance of cathelicidin genes in the liver and spleen between healthy musk deer and those who died of abscess disease. The expression patterns of the six cathelicidins were all upregulated in these two tissues from purulent forest musk deer, except for *CATHL4* in the lung (Figure 6). The marked expression patterns of cathelicidins between the two types of tissues suggest a possible involvement of musk deer cathelicidins in the immune response to abscesses.

## 4. Discussion

Although multiple cathelicidin peptides with a large range of structures have been characterized in cetartiodactyla, limited sequences can be retrieved from species other than food animals. In the present study, we reported the repertoire of forest musk deer cathelicidin genes for the first time. The results of sequence alignment, neighbor-joining tree and genomic structure collectively suggested that all of the cathelicidin genes in the forest musk deer, including the pseudogenes, have already emerged before the divergence of moschidae and bovidae. Similar to previous reports [19,35], the phylogenetic analysis performed in the present study indicated that a series of genes that have high similarity to *CATHL3* are located in the cattle, sheep, and forest musk deer. In addition, we observed independent pseudogenization in the genomes of cattle and forest musk deer. In other words, *CATHL1* lost its function in the forest musk deer, while the orthologous genes of forest musk deer *CATHL3L2* and *CATHL9* became pseudogenes in the cattle. For the *CATHL8p*, a two-exon pseudogene identified both from the forest musk deer and cattle is highly likely to have been fixed in the genome before the two species diverged. However, because only the forest musk deer *CATHL8p* contains a premature stop codon and the two genes are not clustered in the same subclade, we cannot exclude a pseudogenization or partial gene copy event that may have independently occurred in the genome of cattle and forest musk deer. Further genomic investigations in other species are needed to provide detailed evolutionary processes. Given the high sequence similarity presented among the *CATHL3* paralogs, we speculated that the pseudogenization of some cathelicidin genes might be caused by functional redundancy. Interestingly, we noticed that the first intron of *CATHL1p* and *CATHL4* are approximately 110 bp in length (Table 2), which are considerably shorter than the others. Along with the close location of these genes in the genome, these findings suggest that *CATHL1* and *CATHL4* have closely phylogenetic relationships and might diverge from a common ancestor. However, the mature peptides of *CATHL1* and *CATHL4* share a low level of similarity, while both topology of the phylogenetic tree in this research, as well as in other studies [19], indicate that CATHL1 tends to be grouped with CATHL5, and CATHL4 shares more similarity with CATHL2. It has been suggested that in addition to amino acid substitutions driven by positive selection, multiple kinds of evolutionary processes may be involved in the emergence of the distinct cathelicidin subfamilies [36]. For instance, both the protegrins and prophenins in pigs are evolved from a proline-arginine-rich cathelicidin peptide by sequence insertion and internal repeat [37]. It would be particularly interesting to unravel the process that diversified the forest musk deer cathelicidins after gene duplication by using new strategies.

In accordance with previous studies [19], relatively abundant transcripts of many cathelicidin mRNA were detected in the spleen and lung of forest musk deer by RT-PCR. However, we noticed that the expression of cattle *CATHL3* and forest musk deer *CATHL3L2* are largely different. The cattle *CATHL3* was not reliably detected in the spleen [19], whereas a strong expression of *CATHL3L2* was detected in the spleen of forest musk deer. This result could be attributed to the phylogenetic relationship of CATHL3s in these two species. As suggested in this research, the two genes are paralogs instead of orthologs, although they may have been duplicated from a common ancestor. However, given that the samples used for tissue expression analysis were obtained from three individuals that died of natural disasters of different ages and that the mRNA abundance of cathelicidins could be influenced by the living environment, we cannot exclude the possibility that the abundant expression level of *CATHL3L2* in the spleen of forest musk deer is due to raising conditions, as well as other factors. Therefore, additional evidence is needed to confirm our observations in the future. On the other hand, our results showed that the expression of *CATHL4* is decreased in the purulent lung, which was the opposite of the trend observed for the other five cathelicidins. Because several bacterial pathogens have evolved the ability to downregulate HDP expression to better establish themselves in the host [38], the downregulation of *CATHL4* in the lung might be derived from the same mechanism.

Multiple studies have suggested that abscesses are the most prevalent causes of death in the captive population of forest musk deer [6,39,40]. As a widespread and common practice aiming to fight against infection, antibiotics have been routinely used in this species for decades. As a result, bacteria with antibiotic resistance genes have also been identified from captive forest musk deer. For instance, *Arcanobacterium pyogenes*, an opportunistic pathogen that is capable of causing surface or internal abscesses, has been found to have high rates of resistance to β-lactams or trimethoprim in the Miyaluo forest musk deer farm [6]. Alternatives to antibiotics are therefore urgently needed to ensure health and minimize the development of antimicrobial resistance in forest musk deer. Given that HDPs exert pleiotropic effects on both innate and adaptive immune responses, they are being developed into drugs that not only act directly against pathogens but also boost the immune system in unique ways [41]. In addition, because the cationicity of HDPs allows them to blind and kill negatively charged microorganisms through disruption of cell membranes and interaction with intracellular macromolecules, it is very difficult for microbes to develop resistance to HDPs [42]. Although lacking conceivable evidence on the antimicrobial and immune regulatory activities of the forest musk deer cathelicidin peptides, a large number of their orthologous and paralogous peptides from cattle and sheep have been found to be active against microorganisms and to possess immunomodulation activity [43,44]. The peptides thus appear to have the ability to be developed as antimicrobial agents to treat purulent diseases, as well as to combat the growing threat from antimicrobial resistance in captive populations. Alternatively, natural compounds that have the ability to boost endogenous cathelicidin synthesis may also provide a strategy for fighting infectious diseases and alleviating the negative effects caused by the use of antibiotics [45,46,47,48,49,50]. Further exploration of the in vivo and in vitro activities of these peptides may lead to better utilization of these peptides for therapeutic use.

## 5. Conclusions

In conclusion, we identified six functional genes and two pseudogenes from the forest musk deer genome for the first time. Evolutionary analyses indicated that all eight genes have already been fixed in the genome of forest musk deer before the split of moschidae and bovidae, and independent pseudogenization events have occurred after species divergence. On the other hand, the results of sequence comparison, net charge and expression pattern analyses showed a crucial role of forest musk deer cathelicidins in the immune system. These results have a potential application to combat infections with a low risk of developing resistance. This study increased our understanding of the cathelicidins and will be useful for further genetic studies, as well as for the discovery of novel antimicrobial agents in the future.

## Figures and Tables

**Table 1:** Primer sequences used for real-time PCR analysis.

Gene	Forward Primer	Reverse Primer	Fragment Length (bp)	Annealing Temperature (°C)
*CATHL1p*	CTACAGGGACGCCGTGCTTT	GGGCTTTCTGGGTCTTCATCAT	120	60
*CATHL3L2*	CAGAGCAATGTGACTTCAAGGAGA	GGTCGTTTAGCCCTGACACTCTG	127	60
*CATHL4*	CCCGGAGCAGTGTGACTTCAA	CTGGTCATTGGACGGGTTCAG	82	60
*CATHL5*	GGTCGGGAGTAACTTCGACATTAC	GGCCATACCTCTTCCAACCAT	101	60
*CATHL6*	CCAAGGACGATGAGAACCCAA	GTCCAGAGTGACTGTCCCCACA	152	60
*CATHL7*	CCCAGGCCCTCAGCTACAGT	CTCACAGGCTTTCGAGCACCA	148	60
*CATHL9*	GGGGAACTCGAAAGCCTGTGA	CACACTGTTTTACCAGCCCATTCT	114	60
*GAPDH*	GCAAGTTCAACGGCACAGTCA	CTGGTTCACGCCCATCACAA	249	60

**Table 2:** Gene and genomic organizations of forest musk deer cathelicidins.

Gene	Scaffold	Gene Size (bp) ^1^						
		E 1	I 1	E 2	I 2	E 3	I 3	E 4
*CATHL1p*	Scaffold41	198	115	107	150	72	561	117
*CATHL3L2*	Scaffold511	198	624	108	157	72	602	171
*CATHL4*	Scaffold511	198	106	108	137	72	603	66
*CATHL5*	Scaffold511	201	611	108	163	72	597	102
*CATHL6*	Scaffold511	201	614	108	139	72	600	99
*CATHL7*	Scaffold41	201	620	108	154	72	600	117
*CATHL8p*	Scaffold511	198	579	108	NA	NA	NA	NA
*CATHL9*	Scaffold511	198	623	108	165	72	601	111

^1^ Each intact cathelicidin gene consists of four exons (E) separated by three introns (I). The sizes of the exons were predicted based on the deduced amino acid sequences.

## Supplementary Materials

The following are available online at https://www.mdpi.com/2076-2615/9/8/481/s1, Table S1: Locations and orientations of the forest musk deer cathelicidin genes, File S1: Sequence alignments of the cathelicidins used to create the neighbor-joining tree.
